# Highly selective CO_2_ photoreduction to CO on MOF-derived TiO_2_†

**DOI:** 10.1039/d2su00082b

**Published:** 2023-02-17

**Authors:** Matthew Garvin, Warren A. Thompson, Jeannie Z. Y. Tan, Stavroula Kampouri, Christopher P. Ireland, Berend Smit, Adam Brookfield, David Collison, Leila Negahdar, Andrew M. Beale, M. Mercedes Maroto-Valer, Ruaraidh D. McIntosh, Susana Garcia

**Affiliations:** a Research Centre for Carbon Solutions, School of Engineering and Physical Sciences, Heriot-Watt University EH14 4AS UK s.garcia@hw.ac.uk; b Laboratory of molecular simulation (LSMO), Institut des Sciences et Ingénierie Chimiques, Ecole Polytechnique Fédérale de Lausanne (EPFL) Rue de l’Industrie 17 CH-1951 Sion Switzerland; c Department of Chemistry, The University of Manchester Oxford Road Manchester M13 9Pl UK; d Department of Chemistry, University College London 20 Gordon Street London WC1H 0AJ UK; e Catalysis Hub, Research Complex at Harwell, Rutherford Appleton Laboratory Harwell Oxfordshire OX11 0FA UK; f Institute of Chemical Sciences, School of Engineering and Physical Sciences, Heriot-Watt University EH14 4AS UK r.mcintosh@hw.ac.uk

## Abstract

Metal–Organic Framework (MOF)-derived TiO_2_, synthesised through the calcination of MIL-125-NH_2_, is investigated for its potential as a CO_2_ photoreduction catalyst. The effect of the reaction parameters: irradiance, temperature and partial pressure of water was investigated. Using a two-level design of experiments, we were able to evaluate the influence of each parameter and their potential interactions on the reaction products, specifically the production of CO and CH_4_. It was found that, for the explored range, the only statistically significant parameter is temperature, with an increase in temperature being correlated to enhanced production of both CO and CH_4_. Over the range of experimental settings explored, the MOF-derived TiO_2_ displays high selectivity towards CO (98%), with only a small amount of CH_4_ (2%) being produced. This is notable when compared to other state-of-the-art TiO_2_ based CO_2_ photoreduction catalysts, which often showcase lower selectivity. The MOF-derived TiO_2_ was found to have a peak production rate of 8.9 × 10^−4^ μmol cm^−2^ h^−1^ (2.6 μmol g^−1^ h^−1^) and 2.6 × 10^−5^ μmol cm^−2^ h^−1^ (0.10 μmol g^−1^ h^−1^) for CO and CH_4_, respectively. A comparison is made to commercial TiO_2_, P25 (Degussa), which was shown to have a similar activity towards CO production, 3.4 × 10^−3^ μmol cm^−2^ h^−1^ (5.9 μmol g^−1^ h^−1^), but a lower selectivity preference for CO (3 : 1 CH_4_ : CO) than the MOF-derived TiO_2_ material developed here. This paper showcases the potential for MIL-125-NH_2_ derived TiO_2_ to be further developed as a highly selective CO_2_ photoreduction catalyst for CO production.

## Abstract 2

Metal–Organic Framework (MOF)-derived TiO_2_, synthesised through the calcination of MIL-125-NH_2_, is investigated for its potential as a CO_2_ photoreduction catalyst.

## Graphical abstract

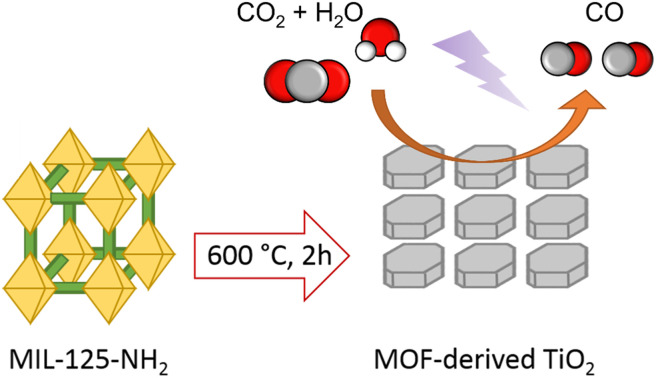

**Sustainability spotlight statement**

Historically, most of our daily chemicals have been synthesised from syngas (CO & H_2_) *via* the Fischer–Tropsch process. Fossil fuels are the main carbon-based feedstocks in the process, making them responsible for a large amount of the GHG emissions from the chemical sector. Alternative renewable and sustainable sources of CO and H_2_ would allow for the development of fossil fuel-free and carbon neutral chemicals and products without replacing existing infrastructure. This work investigates the potential of a MOF-derived TiO_2_ photocatalyst for CO_2_ photoreduction that is selective for CO production. This reaction utilises light, water and CO_2_ and offers a sustainable route to CO production. Hence, this work aligns with several UN SDGs, namely, 7, 12, and 13.

## Introduction

The increased pressure on governments and industries to commit to sustainable energy and production practices has generated a lot of interest in technologies that can address greenhouse gas emission reduction targets.^1^ Carbon Capture Utilisation and Storage (CCUS) is one of those technologies, where the utilisation of CO_2_ has the potential double benefit of not only reducing greenhouse gas emissions but also providing a financial incentive to do so through the production of commodity chemicals.^2–8^ Many different approaches have been trialed in order to convert CO_2_ to fuels or other value-added products, including catalytic, electrocatalytic, photocatalytic and photoelectrocatalytic reactions.^9–16^ In each of these cases, the high stability of the C=O bond (Δ*G*° = −394.36 kJ mol^−1^) and linear geometry of CO_2_ poses a thermodynamic challenge, which needs to be overcome before it can be transformed to valuable compounds. Photocatalytic processes offer a low cost and carbon neutral pathway to overcoming these challenges, as sunlight will be the primary energy source to convert CO_2_ into fuels or other high-value hydrocarbons. Of note is the conversion of CO_2_ to form methane and carbon monoxide, as the former can feed into already established energy systems and the latter can act as a sustainable C1 building block for several products derived from Fischer–Tropsch processes, when partnered with a sustainable source of hydrogen.

The success of any photocatalytic reaction relies on the efficiency of the photocatalysts available. There are three critical steps that govern the efficiency of a photocatalyst; (1) light absorption to generate charge carriers (electrons (e^−^) and holes (h^+^)), (2) charge separation and migration of charges to the active sites, and (3) the redox reaction itself.^17,18^ Each of these steps is heavily influenced by the physical and electronic properties of the photocatalyst, with improvements made in any of the steps being impactful on the overall performance.^19^ There are a number of materials that have been used as photocatalysts for CO_2_ photoreduction *e.g.*, semiconductors, Metal–Organic Frameworks (MOFs), and organometallic complexes.^18–26^ Semiconductors are the most prominent class of photocatalysts, with TiO_2_ being the most studied of these due to its high photostability, low cost, high natural abundance and low toxicity.^27^ Since its first demonstrated use, in water splitting by Fujishima and Honda in 1972, TiO_2_ has been studied extensively and has found success as a catalyst for CO_2_ photoreduction.^27–30^ The latest state of the art TiO_2_ based photocatalysts focus on minimising the charge recombination to maximise their efficiency, and there are a number of strategies that are employed to achieve this.^31–33^ One such method is to use the pores of a MOF as a chamber to grow TiO_2_ particles.^34^ This allowed for synergy between the light absorbing/electron generating TiO_2_ and the catalytic metal clusters of the MOF to enhance CO_2_ reduction.^34^ Noble metal co-catalysts are commonly introduced as a method to enhance light absorption as well as reducing electron–hole recombination.^35,36^ Another method, described in Angulo-Ibáñez *et al.*'s work involves the synthesis of a TiO_2_ based metal–organic aerogel which results in a highly active photocatalyst for methanol production.^37^ These methods focus on improving light conversion to charge carriers and prioritise facilitating the photoreduction reaction. Great improvements have been made in these areas, however, the lack of selectivity in the products produced is still a challenge that has yet to be overcome.

Previous work by Kampouri *et al.* prepared mixed-phase TiO_2_ nanoparticles through calcination of a MOF, MIL-125-NH_2_, and showcased enhanced activity for hydrogen production *via* photocatalytic water splitting.^38^ The MOF structure acted as a sacrificial template to form rectangular parallelepipeds particles of TiO_2_ as the organic components were burned away. Through strict control of the calcination temperature they were able to alter the ratio of anatase and rutile in the resultant TiO_2_ nanoparticles. Subsequent study of the MOF-derived TiO_2_ for photocatalytic hydrogen generation was promising with the material exhibiting high H_2_ production rates outperforming P25 (Degussa) – a common commercial TiO_2_ benchmark. They attribute the excellent performance of the MOF-derived TiO_2_ the formation of longer lived charge carriers due to reduced electron–hole recombination afforded by charge transfer between the anatase and rutile phases in the TiO_2_ nanoparticles within the MOF-templated crystals. Given the reduced electron–hole recombination and enhanced activity, compared to P25 (Degussa), this material looks to be well suited to catalysing CO_2_ photoreduction. As such, this work examines the potential of this material as a photocatalyst for CO_2_ reduction, exploring the impact of temperature, irradiance, and partial pressure of water (*P*_H_2_O_) on CO_2_ conversion.

## Experimental

### Photocatalyst preparation

The MOF derived TiO_2_ was synthesised according to work of Kampouri *et al.*, the details can be found in the ESI† along with the characterisation used to confirm its successful synthesis (SF. 1†).^38^

Once acquired, the MOF-derived TiO_2_ was loaded onto a glass fiber disc support using the following protocol: approximately 10 mg of the photocatalyst was added to 1 mL of ultrapure water in a 10 mL glass sample vial. The suspension was then agitated in an ultrasonic bath for 3 minutes and the resultant slurry was applied to a 47 mm glass fiber disc (Whatman) by drip coating before drying in an oven at 120 °C for two hours. The same protocol was followed using a commercial P25 photocatalyst, which was used for benchmarking purposes.

### Photoreduction test

**Fig. 1:**
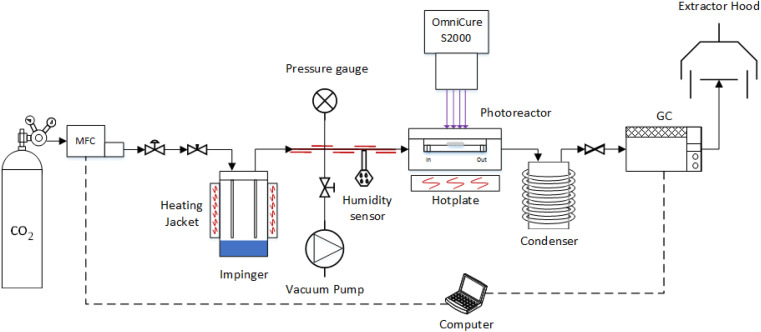
Overview of the experimental setup used for the CO_2_ photoreduction tests (Not to scale).^34^

The photoreduction of CO_2_ was investigated in a purpose-built gas phase system (Fig. 1) using water as the reducing reagent, used previously by our group.^39,40^ The photocatalyst coated glass fibre disc was placed in the middle of the photoreactor and, to eliminate residual air contamination, the system was evacuated *via* three swing purge-vacuum steps placing the system under vacuum and then purging with CO_2_ (99.995%). The flow rate of CO_2_ was set to 0.35 mL min^−1^ and was passed through the temperature controlled (±0.1 °C) aluminium body saturator for at least 16 h to allow the system to equilibrate. To record the partial pressure of H_2_O, relative humidity (±1.8% RH) was measured using an inline Sensirion SHT75 humidity sensor placed (MG Chemicals 832HD) into a Swagelok 1/4′′ T-piece. The photoreactor was heated using a hotplate and after at least 16 h equilibration, the surface temperature of the coated photocatalyst was measured using a Radley's pyrometer (±2.0 °C). To prevent condensation at higher relative humidity, the lines from the outlet of the impinger, up until the inlet of the H_2_O trap, were heated and temperature controlled (±0.1 °C) with a heating cord and thermocouple (Fig. 1).

An OmniCure S2000 (300–600 nm) was used as the light source and the irradiance set according to the experimental design (Table 1). The light source was placed 30 mm above the surface of the coated glass fibre disc and irradiance, at the exit of the fibre optic light guide, was measured before each experiment by using an OmniCure R2000 radiometer (±5%). An inline GC (Agilent, Model 7890B series) with a Hayesep Q column (1.5 m), 1/16 inch od, 1 mm id, MolSieve 13X (1.2 m), 1/16 inch od, 1 mm id, thermal conductivity detector (TCD), nickel catalysed methanizer and flame ionization detector (FID) was used to analyse the output of the photoreactor every four minutes. The GC was calibrated using 1000 ppm calibration gas (H_2_, CO, O_2_ and CH_4_) in a balance of Ar gas that was further diluted with Ar (99.995%). Cumulative production (μmol cm^−2^) was calculated by integrating the area under the production rate (μmol cm^−2^ h^−1^) *vs.* time (h) curve using the trapz MATLAB function.

**Table 1:** Full factorial design used for investigating experimental space of irradiance, temperature and partial pressure of H_2_O for MOF-derived TiO_2_. Where −1, 0, and 1 represent the low, intermediate and high settings of the explored experimental conditions, respectively

Std order	Irradiance	Temperature	*P*_H_2_O_	Irradiance (W m^−2^)	Temperature (°C)	*P*_H_2_O_ (kPa)
Exp 1	−1	−1	−1	1480	38.4	2.48
Exp 2	−1	−1	1	1520	37.5	3.02
Exp 3	−1	1	−1	1490	55.6	2.49
Exp 4	−1	1	1	1490	56.3	3.14
Exp 5	1	−1	−1	2980	38.2	2.34
Exp 6	1	−1	1	2870	38.1	3.05
Exp 7	1	1	−1	2890	56.1	2.41
Exp 8	1	1	1	2850	56.1	3.02
Exp 9	0	0	0	2240	46.1	2.72
Exp 10	0	0	0	2230	45.0	2.79
Exp 11	0	0	0	2230	46.0	2.83

### Design of experiments

The impact of irradiance, temperature, and partial pressure of H_2_O on photoreduction was tested for the MOF-derived TiO_2_ photocatalyst within the following experimental ranges: irradiance (150–300 mW cm^−2^), temperature (38–56 °C) and *P*_H_2_O_ (2.50–3.00 kPa). In order to properly explore the large experimental space with the minimum number of experiments a two-level full-factorial experimental design with three central points was used to systematically investigate the experimental space shown in Table 1. Additional details of the experimental design can be found in the ESI.†

To allow for a more appropriate comparison to other systems and photocatalysts, additional photoreduction experiments were carried out using the best performing conditions and a commercially available P25 (Degussa) sample, as a reference material.

## Results and discussion

**Fig. 2:**
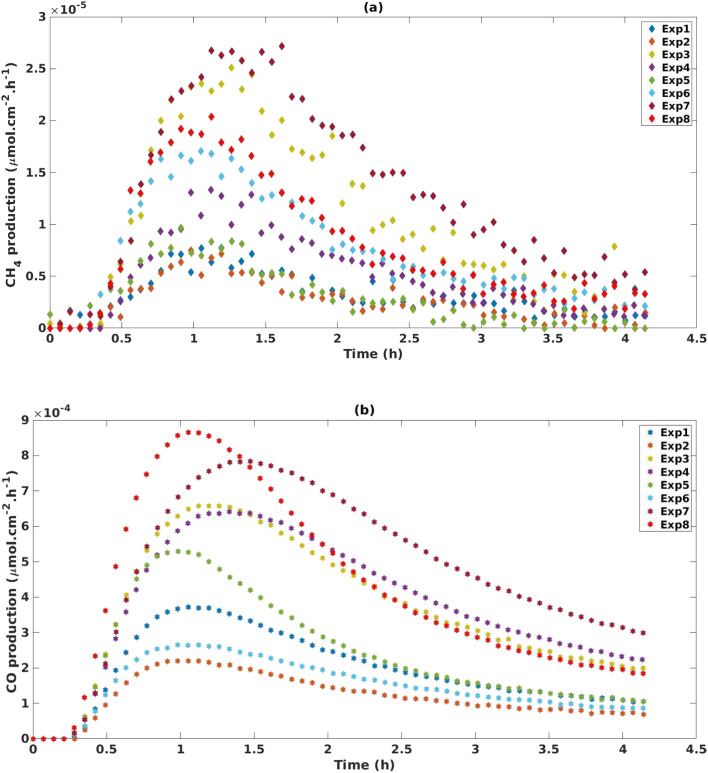
(a) CH_4_ and (b) CO production using full-factorial design experimental setting points.

The experimental space investigation in Table 1 revealed that MOF-derived TiO_2_ produced both, CH_4_ and CO (Fig. 2), with a significant selectivity towards CO production which was, on average, an order of magnitude greater than CH_4_ production. Both CH_4_ and CO production profiles peak between 1–1.5 hours before exhibiting a deactivation profile. This deactivation trend is a common observation for TiO_2_ based catalysts for CO_2_ photoreduction and may be attributed to a loss of oxygen vacancies or accumulation of reaction intermediates on the catalyst surface.^41–44^ The reproducibility and consistency of our experimental design was confirmed through triplicate tests at the central points (SF. 8†). For CH_4_, the three central points yielded an average cumulative production of 3.70 × 10^−4^ μmol cm^−2^ ± 1.39 × 10^−4^ μmol cm ^−2^. For CO production the central points yielded good reproducibility with an average cumulative production of 1.85 × 10^−2^ μmol cm^−2^ ± 2.46 × 10^−3^ μmol cm^−2^. The conditions used in Exp 8 (Table 1) were found to have the greatest production rate of CO, peaking at a rate of 8.9 × 10^−4^ μmol cm^−2^ h^−1^ (2.6 μmol g^−1^ h^−1^) and cumulatively producing 2.1 × 10^−2^ μmol cm^−2^ (64.0 μmol g^−1^) of CO over 4 h. Exp 7 was found to have the greatest production rate of CH_4_ peaking at 2.6 × 10^−5^ μmol cm^−2^ h^−1^ (0.1 μmol g^−1^ h^−1^) and cumulatively producing 6.28 × 10 μmol cm^−2^ (2.5 μmol g^−1^ h^−1^) of CH_4_.

**Fig. 3:**
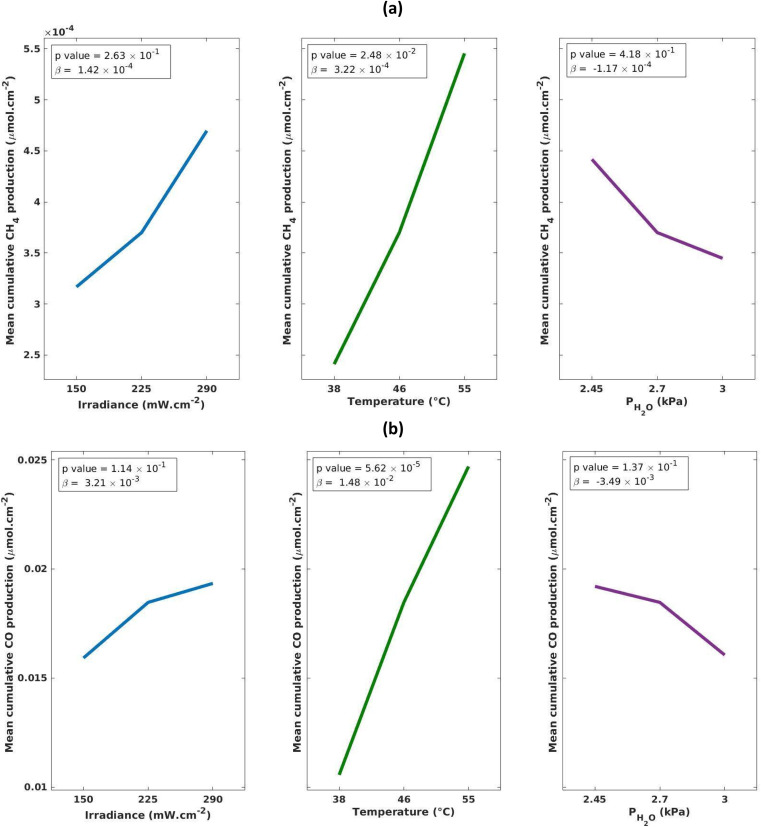
(a) CH_4_ and (b) CO main effects plots to visually highlight the strength of each reaction parameter.

The results of the DoE shown in Fig. 3 indicate that for cumulative production of both CH_4_ and CO, temperature is the only statistically significant parameter. This can also be observed in Fig. 2b where Exp 3, 4, 7 and 8, which have the highest temperature settings in the design, exhibit increased production of CO. The increase in performance with increasing temperature has been documented in a number of studies, wherein it is believed to contribute to the initial breaking of C–O bonds in CO_2_ adsorbed to the surface of TiO_2_.^45–47^ For instance, Liu *et al.* report a 10-fold increase in performance when the temperature was gradually increased from 50 to 150 °C. However, there does appear to be an optimum temperature window with further increases to 170 °C showing a decrease in activity.^43^ In a separate study, it is proposed that elevated temperatures aid in the desorption of intermediates and products making the active sites available for CO_2_ adsorption.^48^

In this work, the range of explored irradiance values (1480–2980 W m^−2^) displayed no statistically significant impact on the production of CO or CH_4_ (Fig. 3). This is consistent with work by Dilla *et al.* which examines the irradiance relationship in P25 (Degussa) and finds that at high irradiances (230–1700 W m^−2^) there is a minimal increase in the rate in rate of production, whereas at low irradiances (<230 W m^−2^) there is a much larger increase in the rate of production, although the overall rate remains low.^49^ The authors explain that at higher irradiances the higher concentration of charge carriers lead to an increase in charge transfer to adsorbed species, but also increase the thermodynamically preferred charge-recombination events hence leading to an overall minimal increase in the rate. Similarly, the work by Olivo *et al.* also finds that low irradiance (40–60 W m^−2^) is a statistically significant parameter, attributing this to an insufficient number of photons to activate all available photocatalytic sites.^39^ However, they find that at high irradiances (60–2400 W m^−2^) there is a minor dependence on irradiance for CH_4_ production, attributing this to fewer incident photons being required to activate the photocatalytic sites. These reports are consistent with our findings and indicate that the active sites on the MOF-derived TiO_2_ are also fully saturated at irradiances as low as 1480 W m^−2^, and likely even as low as 230 W m^−2^. This is notable for potential commercialization of this photocatalyst, as the average solar irradiance on earth's surface is ∼325 W m^−2^ of which ∼8% is made up of UV light, meaning that solar concentrators would be required to artificially boost irradiance if the light source being used is natural solar light.^50,51^ This is also highly dependent on the location and the set-up of any future plant as surface solar irradiance varies significantly based on location, for example in the UK the highest average annual solar irradiance was in the range of 123.4–126.0 W m^−2^.^52^

The range of H_2_O partial pressures (2.48–3.05 kPa) explored here do not have a statistically significant relationship with CO/CH_4_ production (Fig. 3). The effect of H_2_O is important to monitor as it is an integral parameter to facilitate the reaction. Without the presence of water the reaction cannot proceed, however, at elevated levels it has been shown to inhibit the reaction due to blocking the active sites or weakening CO_2_ adsorption.^42^ Previous investigations by others into the effect of water concentration on the production of CH_4_/CO give contradictory results. Molins *et al.* find a statistically significant increase in photocatalytic production of methane with increasing water mole fraction (*x*_H_2_O_ = 0.25–0.75) across Pt/TiO_2_ (P25) photocatalysts, whereas Dilla *et al.* found that a continuous flow of H_2_O inhibited CH_4_ formation due to competition with CO_2_ over active sites.^47,49^

**Fig. 4:**
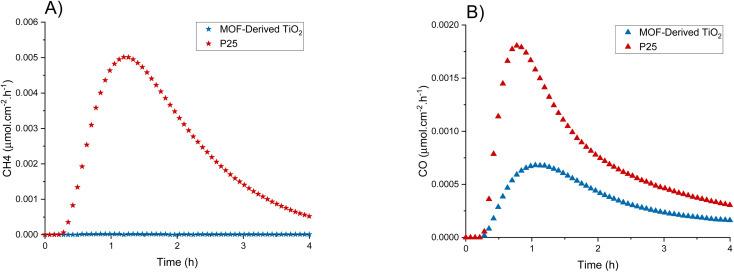
MOF-derived TiO_2_ and P25 production rates of (A) CH_4_ and (B) CO using the best performing conditions found from the design of experiment (Table 1, Exp 8).

To allow for an appropriate comparison to other systems and photocatalysts, additional photoreduction experiments were carried out using the conditions in Exp 8 and the commercially available P25 (Degussa) as a reference material (Fig. 4). P25 exhibits a greater overall activity towards CO_2_ photoreduction than the MOF-derived TiO_2_, producing appreciable amounts of both CO and CH_4_, and reaching an average maximum production rate of 1.4 × 10^−3^ μmol cm^−2^ h^−1^ (2.5 μmol g^−1^ h^−1^) and 5.0 × 10^−3^ μmol cm^−2^ h^−1^ (8.9 μmol g^−1^ h^−1^) for CO and CH_4_, respectively. P25 cumulatively produced 0.044 μmol cm^−2^ (0.078 μmol g^−1^) and 0.14 μmol cm^−2^ (0.24 μmol g^−1^) on average for CO and CH_4_, respectively. Both photocatalysts follow similar deactivation profiles after peaking at ∼1 h. The selectivity of P25 leans towards CH_4_ (3 : 1 CH_4_ : CO) which is in accordance with literature.^41,53–55^ Conversely, MOF-derived TiO_2_ has almost complete selectivity towards CO (1 : 49 CH_4_ : CO) albeit with a lower total activity. This lower activity was unexpected as the MOF-derived TiO_2_ was shown to have a longer charge lifetime when compared to P25, which is an integral part of the photoreduction process therefore we were anticipating enhanced activity.^38^

It is an interesting and important observation that the MOF-derived TiO_2_ produces almost exclusively CO. There are several factors that could cause this; the surface of the photocatalyst could be more prone to desorbing CO intermediates, or the reduced surface area of the MOF-derived TiO_2_, when compared to P25, results in fewer surface bound hydroxyls available to participate in the reaction.^38^ Typically, purely TiO_2_ based semiconductors have exhibited poor control over the products of the photoreduction leading to inefficient reactions. The selectivity of photocatalysts is strongly influenced by the surface of the material and how it interacts with adsorbed species.^18^ For example, in a work by Li *et al.* the introduction of dual-metal sites shifted the selectivity to almost 100% CH_4_ through stabilisation of C–O intermediates preventing their desorption prior to reduction.^56^

**Fig. 5:**
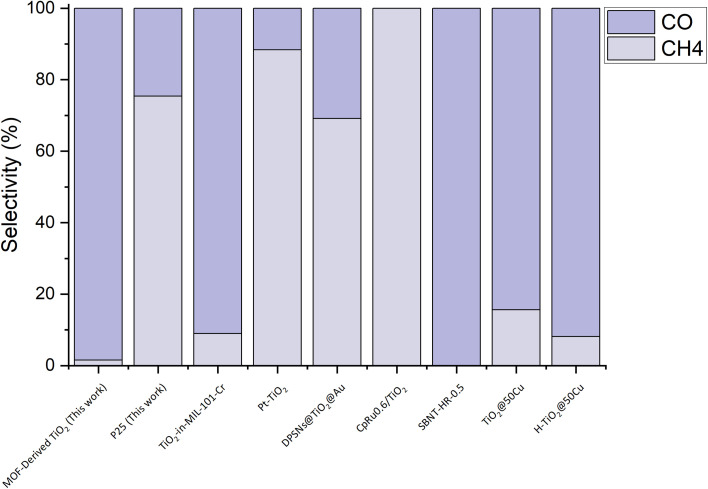
Comparison of the selectivity of the MOF-derived TiO_2_ (Exp 8) compared to P25 and other state-of-the-art CO_2_ photoreduction catalysts.

When comparing MOF-derived TiO_2_ to other state-of-the-art CO_2_ photocatalysts, in terms of selectivity, the potential of this material is highlighted (Fig. 5).^34–36,57–59^ Being able to achieve a 98% selectivity towards CO without a co-catalyst is very significant, as typically TiO_2_ photocatalysts are coupled with expensive noble metal co-catalysts to alter the surface in such a way as to direct the selectivity.^30,60^ When compared to other photocatalysts (Fig. 5), the MOF-derived TiO_2_ is only outcompeted in selectivity by the molecular photocatalyst grafted to TiO_2_, CpRu_0.6_/TiO_2_, and SBNT-HR-0.5, Sr_2_Bi_2_Nb_2_TiO_12_ nanosheets, which are 100% selective for CH_4_ or CO, respectively (experimental and production details can be found in S3 of the ESI†).^57,58^ When taking into consideration the relatively simple synthesis and abundance of the required materials, MOF-derived TiO_2_ is an attractive catalyst for CO_2_ photoreduction. Currently there are areas for improvement/development before this photocatalyst can match the other state of the art catalysts. For instance, the activity of the MOF-derived TiO_2_ is quite low compared to current top performers, such as those reported by recent work from Jiang *et al.*, where they observe a production rate of 12 mmol g^−1^ h^−1^ without a decay in performance across 60 h.^34^ The second area for further development is enhancing the visible-light absorption. MOF-derived TiO_2_ is still a form of TiO_2_ that does not absorb significantly in the visible light region of the solar spectrum, which affects its potential to generate charges to take part in the reaction. However, addressing these issues is quite feasible through further optimisation or modification of the catalyst.

Cycle tests were carried out to investigate the longevity and stability of MOF-derived TiO_2_. These show a decline in performance after two cycles (SF. 9†), which may be due to accumulation of intermediates on the active sites.^42^ The performance may potentially be recovered through regeneration of the catalyst through a heat cycle to desorb the intermediates.

**Fig. 6:**
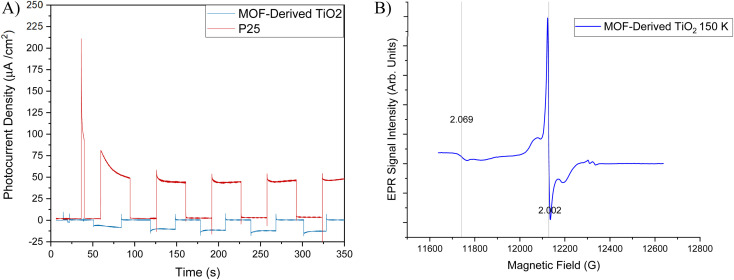
(A) Photocurrent response for MOF-derived TiO_2_ & P25 (B) Q-band continuous wave EPR spectra of the MOF derived TiO_2_ at 150 K. Microwave frequency 34.023535 GHz, microwave power 0.1 mW, modulation amplitude 5 G.

To understand the observed selectivity additional investigations into the electronic properties were conducted. Fig. 6A shows the photocurrent response for MOF-derived TiO_2_ under UV-visible (*λ* = 320–1000 nm) light irradiation at a constant potential 0.5 V (*vs.* SCE). The MOF-derived TiO_2_ exhibits a sharp response to light, reaching a peak photocurrent density of −18.33 μA cm^−2^ before equilibrating at −12.70 μA cm^−2^ before sharply returning to the baseline upon the light being turned off. There is an increase in photocurrent density (−8.51 to −12.70 μA cm^−2^) over time first giving a peak response of −8.51 μA cm^−2^ before gradually increasing to −12.70 μA cm^−2^ where it appears to reach a plateau. This is an interesting result for two reasons, firstly the MOF-derived TiO_2_ shows a lower photocurrent density compared to that of P25, which would contribute to the higher activity shown by P25 towards CO_2_ photoreduction. The second reason is that the MOF-derived TiO_2_ has a negative photocurrent density. This is indicative of p-type semiconductor behaviour, which is typically not observed in undoped TiO_2_-based materials where typically n-type semiconductor behaviour is observed. The p-type behaviour in undoped TiO_2_ species has been suggested linked to Ti vacancies present within the material.^61,62^ To investigate this hypothesis, Electron Paramagnetic Resonance (EPR) spectra were collected to determine if these p-type Ti vacancies were present in the MOF-derived TiO_2_. As reported in the literature, p-type vacancies usually present as relatively strong signals with *g*-values ≈ 1.998 and are typically measurable at both cryogenic temperatures and higher (towards room temperature) in contrast to the anatase/rutile defects present in commercial TiO_2_, where cryogenic temperatures are vital for adequate sensitivity of detection.^63,64^

Variable temperature EPR spectra of the MOF-derived TiO_2_ were collected at both X-band (SF. 3†) (≈9.4 GHz) and Q-band (≈34 GHz) frequencies and confirm the presence of the Ti vacancies with *g* = 2.002 (Fig. 6B). The higher frequency of Q-band was particularly useful to unpack the spectra by separating the resonances of the trace copper impurities (one component is at *g* = 2.069 and is confirmed by XRF, arising from starting materials, SF. 5†) away from the signal of interest. The *g*-value obtained differs slightly to the literature value but is within the range of uncertainties present when carrying out field correction against a standard sample, and the shape of the resonance is as reported previously.^63^

The effect of titanium vacancies on the electronic properties have been reported by Bak *et al.*^65^ They note that charge transfer is enhanced for electrons and decreased for holes, as a result charge recombination is reduced. This phenomenon is observed in MOF-derived TiO_2_ through the photoluminescence experiments carried out in the original work by Kampouri *et al.*, where it exhibits lower emissions compared to P25.^38^ Wang *et al.* therefore attribute the enhancement in organic pollutant degradation performance to these electronic properties arising from the presence of titanium vacancies within the structure of the photocatalyst.^63^ Although, in this work enhanced activity was not observed *vs.* P25, possibly due to the inherent differences between CO_2_ photoreduction reaction and pollutant degradation. To further our understanding of the reaction mechanism *in situ* Diffuse Reflectance Infrared Fourier Transform Spectroscopy (DRIFTS) studies were conducted on P25 and MOF-derived TiO_2_.

**Fig. 7:**
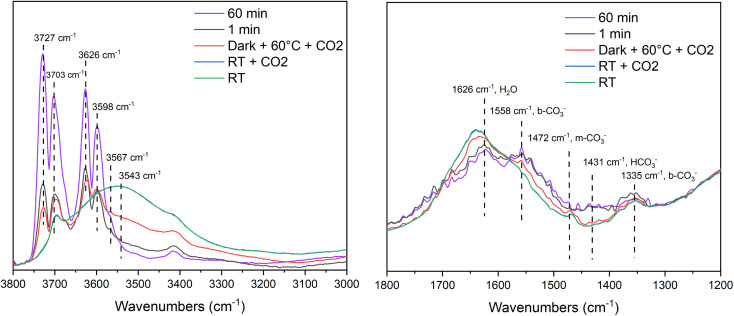
*Operando* DRIFT spectra for CO_2_ photoreduction on MOF-derived TiO_2_ before and after irradiation for 60 minutes.

DRIFT spectra of the MOF-derived TiO_2_ at room temperature, at room temperature with CO_2_, at 60 °C with CO_2_, after 1 minute of light exposure and after 60 minutes of light exposure are shown in Fig. 7. The peaks at 3543 and 1626 cm^−1^ are assigned to H_2_O with the former being broad due to the effects of H-bonding with other molecules or surface hydroxyls. Hydroxyl signals can be observed 3727–3598 cm^−1^, which can be assigned to non-H-bonded “free” hydroxyls that could be found on the edges of MOF-derived TiO_2_ particles.^66^ These signals become more pronounced upon light exposure and temperature increasing. Increasing the temperature from room temperature to 60 °C decreases the signals at 3567 and 3543 cm^−1^, which further suggests that they are a combination of H-bonded molecular water and hydroxyl groups. The CO_2_*ν*_as_ (CO) signal can be found at 2347 cm^−1^ (SF. 6†) which is supported by values found in literature.^67^ The CO signal is broad and is centered around 2048 cm^−1^ which is relativity low compared to literature but still within an appropriate range (2200–2050 cm^−1^).^68–70^ The region from 1800–1200 cm^−1^ is where the mono- and bi-dentate carbonate (1558 *ν*_as_ (OCO) b-CO_3_^2−^, 1472 m-CO_3_^2−^, 1335 b-CO_3_^2−^ cm^−1^), bicarbonate (1431 cm^−1^) and water (1626 cm^−1^) signals are found.^71,72^ The broad nature of the signals in this region makes accurate assignment challenging. However, based on the changes with temperature we can assign the signal at 1626 cm^−1^ to molecular water as it decreases with increasing temperature, and the signal at 1558 cm^−1^ can be assigned to a carbonate species (*ν*_as_ OCO) given the slight increase in signal with temperature and light exposure. Similar absorption bands were observed in the P25 sample (SF. 7†), however the 1800–1200 cm^−1^ region was even more poorly defined, although the same temperature related trends are present.

There were no signals corresponding the presence of formate or methoxy species, which suggests that formic acid and methanol are not formed in the reaction.^73^ It is possible these products are formed and the intermediates are not observed on this reaction timescale. The *operando* DRIFTS study shows the reaction intermediates and pathways that are important for CO_2_ photoreduction to CO over this catalyst. However, based on DRIFTS result, the mechanism for CO formation is suggested to proceed through a reduction of CO_2_^−^ with H^+^ (eqn (1)) or potentially through a disproportionation reaction between two CO_2_^−^ species (eqn (2)).^74,75^

1 CO_2_^−^ + H^+^ + e^−^ → CO + OH^−^

2 CO_2_^−^ + CO_2_^−^ → CO + CO_3_^−^

## Conclusions

This study showcases a promising new photocatalyst for CO_2_ photoreduction. The systematic investigation of the reaction parameters – irradiance, temperature, and partial pressure of water – through a two-level design of experiment (DoE) has shown that only temperature is a statistically significant parameter for CO_2_ photoreduction. The MOF-derived TiO_2_ was found to produce almost exclusively CO as a photoreduction product. The high selectivity we observe is unusual in purely TiO_2_ based photocatalysts and was achieved without the use of a co-catalyst or expensive rare-earth metals. Comparison experiments were carried out with commercially available P25 (Degussa) using the best conditions found through the DoE. This comparison showed that P25 was more active than MOF-derived TiO_2_, however, this activity was split across both CH_4_ and CO (3 : 1) production. Finally, the selectivity of MOF-derived TiO_2_ was compared with other state of the art CO_2_ photocatalysts and the MOF-derived TiO_2_ compared favourably. The selectivity of the MOF-derived TiO_2_ reported here outperforms the majority of the state-of-the-art photocatalysts which employed co-catalysts to enhance performance. The p-type conductivity of the MOF-derived TiO_2_ is in contrast to the n-type conductivity of P25. Our empirical observations suggest that this plays a role in the selectivity observed in the CO_2_ photoreduction, however further experiments will be required to elucidate the mechanism of this reaction. This catalyst is an exciting prospect and will be the subject of future work that will focus on enhancing its activity whilst maintaining its excellent selectivity.

## Author contributions

Matthew Garvin and Warren A. Thompson: conceptualization, data curation, formal analysis, investigation, methodology, project administration, resources, software, validation, visualisation, writing – original draft; Jeannie Z. Y. Tan: investigation, methodology, writing – review & editing; Stavroula Kampouri, Christopher P. Ireland and Berend Smit: conceptualization, resources, writing – review & editing; Adam Brookfield and David Collison: formal analysis, investigation, methodology, resources, validation, visualisation, writing – original draft; Leila Negahdar and Andrew M. Beale: formal analysis, resources, writing – review & editing; Mercedes Maroto-Valer, Ruaraidh McIntosh and Susana Garcia: project administration, resources, writing – review & editing.

## Conflicts of interest

There are no conflicts to declare.

## Supplementary Material

SU-001-D2SU00082B-s001
