# Supplementary material for: Highly selective CO2 photoreduction to CO on MOF-derived TiO2
Source: RSC Sustain. 2023 Feb 17;1(3):494–503. doi: 10.1039/d2su00082b (PMC10193832; doi:10.1039/d2su00082b)
Supplement: SU-001-D2SU00082B-s001 [file SU-001-D2SU00082B-s001.pdf]

Electronic Supplementary Material (ESI) for RSC Sustainability.
This journal is © The Royal Society of Chemistry 2023

Highly selective CO_2 photoreduction to CO on MOF-derived TiO_2

Matthew Garvin,[a] Warren A. Thompson[a], Jeannie Z. Y. Tan[a], Stavroula Kampouri[b], Christopher P. Ireland[b], Berend Smit[b], Adam Brookfield[c], David Collison[c], Leila Negahdar[de], Andrew M. Beale[de], M. Mercedes Maroto-Valer[a], Ruaraidh D. McIntosh[e*], and Susana Garcia[a*]

[a]Research Centre for Carbon Solutions (RCCS), School of Engineering and Physical Sciences, Heriot-Watt University, UK.

[b]Laboratory of Molecular Simulation (LMSO), Institute of Chemical Sciences and Engineering, École Polytechnique Fédérale de Lausanne (EPFL Valais), Rue de l'Industrie 17, 1951 Sion, Switzerland

[c]Department of Chemistry, The University of Manchester, Oxford Road, Manchester M13 9Pl, UK

[d]Department of Chemistry, UCL, 20 Gordon Street, London, WC1H 0AJ, UK

[eUK] Catalysis Hub, Research Complex at Harwell, Rutherford Appleton Laboratory,Harwell, Oxfordshire, OX11 0FA, UK

[f]Institute of Chemical Sciences, School of Engineering and Physical Sciences, Heriot-Watt University, UK.

Table of Contents

S1 Experimental 2

S.1.1 Materials 2
S.1.2 Experimental Protocol 2
S.1.2.1 Synthesis of MIL-125-NH_2 2
S.1.2.2 Synthesis of MOF-derived TiO_2 2
S.1.3 Characterisation 2
S.1.3.1 Powder X-ray diffraction 2
S.1.3.2 Scanning Election Microscopy 2
S.1.3.3 Electron Paramagnetic Resonance Spectroscopy 3
S.1.3.4 DRIFT 5

S2 Design of Experiment 6

S2.1 Further details of Design of Experiment 6
S2.2 Reproducibility 8

S3 Referenced Work

S1 Experimental

S1.1 Materials

2-aminobenzene-1,4-dicarboxylic acid (NH_2-H_2BDC, 99%), titanium isopropoxide ($Ti(OCH(CH_3)_2)_4$, 97%) and ethanol (absolute EMPLURA®) were purchased from Sigma Aldrich. N,N-dimethylformamide (DMF, ≥ 99.5%) and methanol (MeOH, ≥ 99.9%) were purchased from Carl Roth and Fisher Scientific, respectively. The materials were used as received without further purification.

S1.2 Synthetic Procedures

S1.2.1 Synthesis of MIL-125-NH_2: MIL-125-NH_2 was synthesized by following a reported procedure.[24] 0.286 g of NH_2-H_2BDC was dissolved in a mixture of 4.0 mL DMF and 1.0 mL methanol. 0.286 mL of titanium isopropoxide was added in the mixture, which was sonicated for 30 min and then heated up to 120 °C for 72h. The resulting product was washed several times with DMF and methanol and collected via centrifugation.

S1.2.2 Synthesis of MIL-125-NH_2-derived TiO_2: In a typical preparation, 300 mg of the as-synthesized MIL-125-NH_2 powder was placed in a ceramic crucible covered with foil and calcined for 1 h at 600 °C. The obtained product was washed several times with ethanol and water, and then dried at 70 °C for 12 h.

S1.3 Characterization

S.1.3.1 Powder X-ray Diffraction (PXRD) data was collected using a PANalytical MultiCore Empyrean High-Performance X-ray diffractometer at room temperature. The data was acquired using a high flux non monochromated X-ray source leading to the presence of secondary radiation and secondary

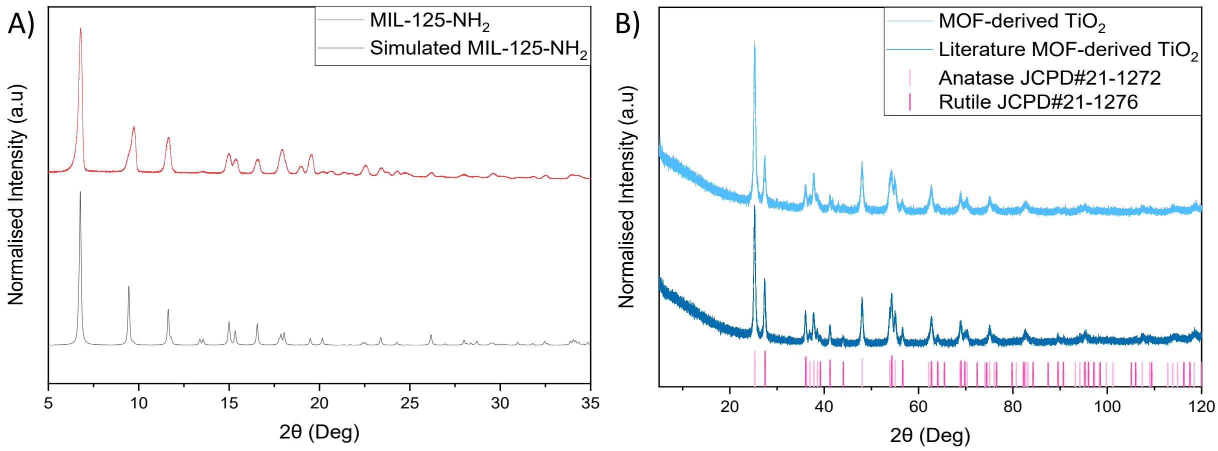

SF 1: *PXRD patterns of A) MIL-125-NH_2 (red) and simulated MIL-125-NH_2 (black) and B) synthesised MOF-derived TiO_2 (light blue) and literature MOF-derived TiO_2 (dark blue)*

peaks.

S.1.3.2 Electron Paramagnetic Resonance Spectroscopy (EPR)

Variable temperature continuous wave EPR measurements were carried out at X-, and Q-band frequencies (9.4, 34 GHz) on a Bruker EMXPlus spectrometer equipped with Bruker ER4112SHQ or ER 5106QT resonator respectively.

Variable temperature measurements were achieved using a Bruker Stinger closed cycle cryocooler mated to an Oxford Instruments ESR900 (X band) or 935CF (Q band) cryostat with temperature control and monitoring handled by an Oxford Instruments MercuryiTC.

Powder samples (of weighed mass) of the MOF derived TiO_2 and the P25 control were prepared in 2 mm (Q-band) and 4 mm (X-Band) Quartz EPR tubes to a matched sample height of 6 cm, designed to enable quantification experiments. g-factor calibration was carried out by comparison to a Bruker strong pitch standard sample with a known g = 2.0028.

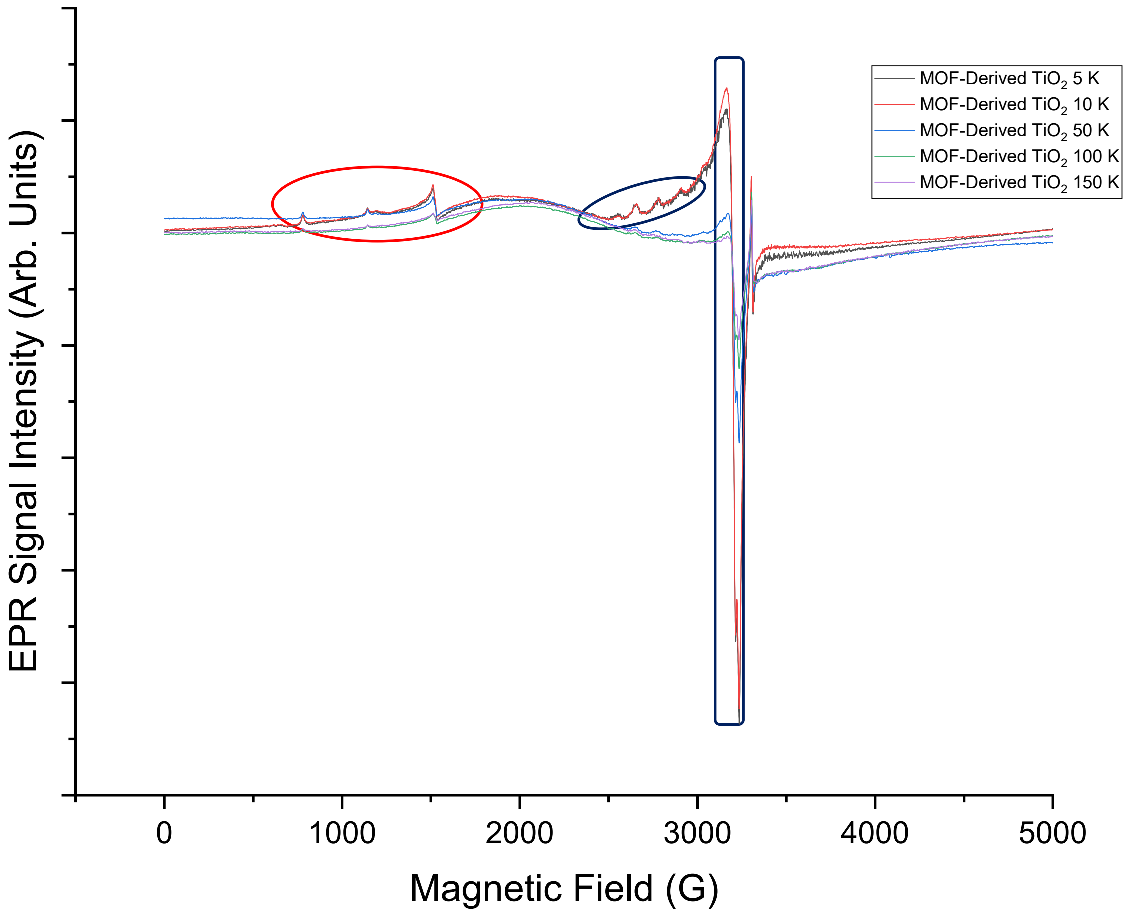

SF 3: *X-Band variable temperature continuous wave spectra of MOF-derived TiO_2. Microwave frequency 9.387 GHz, modulation amplitude 5 G, microwave power 0.2 mW. Spectra show the presence of Fe^{3+} (red outline) and Cu^{2+} (blue outline) impurities along with the effect of temperature versus the defect of interest.*

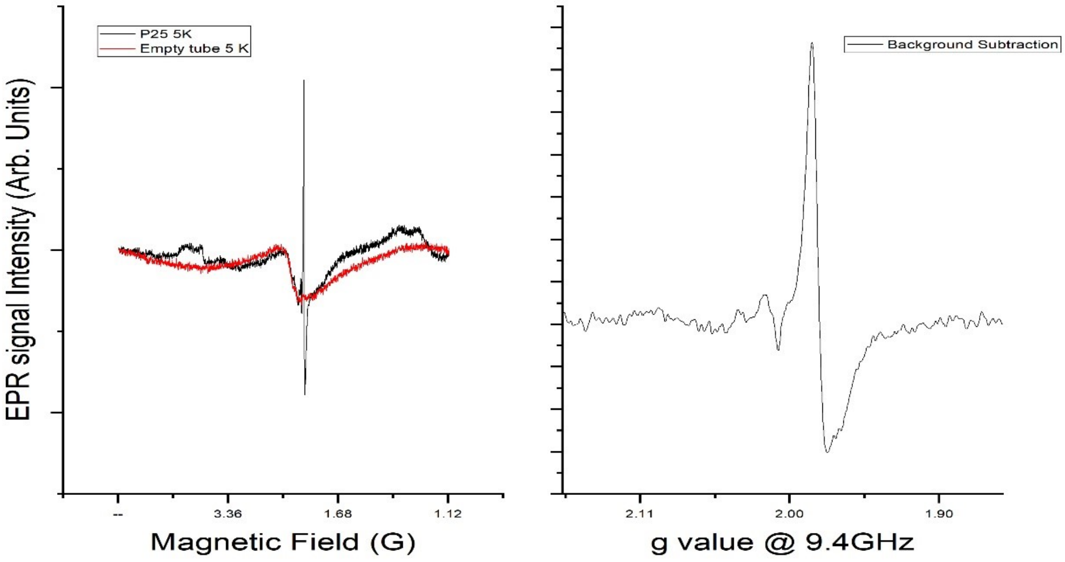

SF 4: *X-Band low temperature continuous wave spectra of Degussa P25. Microwave frequency corrected to 9.4 GHz to allow background subtraction, modulation amplitude 5 G, microwave power 0.2 mW. Spectra show the small relative intensity against the intrinsic* Cu^{2+} *background (from the resonant cavity) and the MOF-derived* TiO_2.

S.1.3.3 X-ray Fluorescence

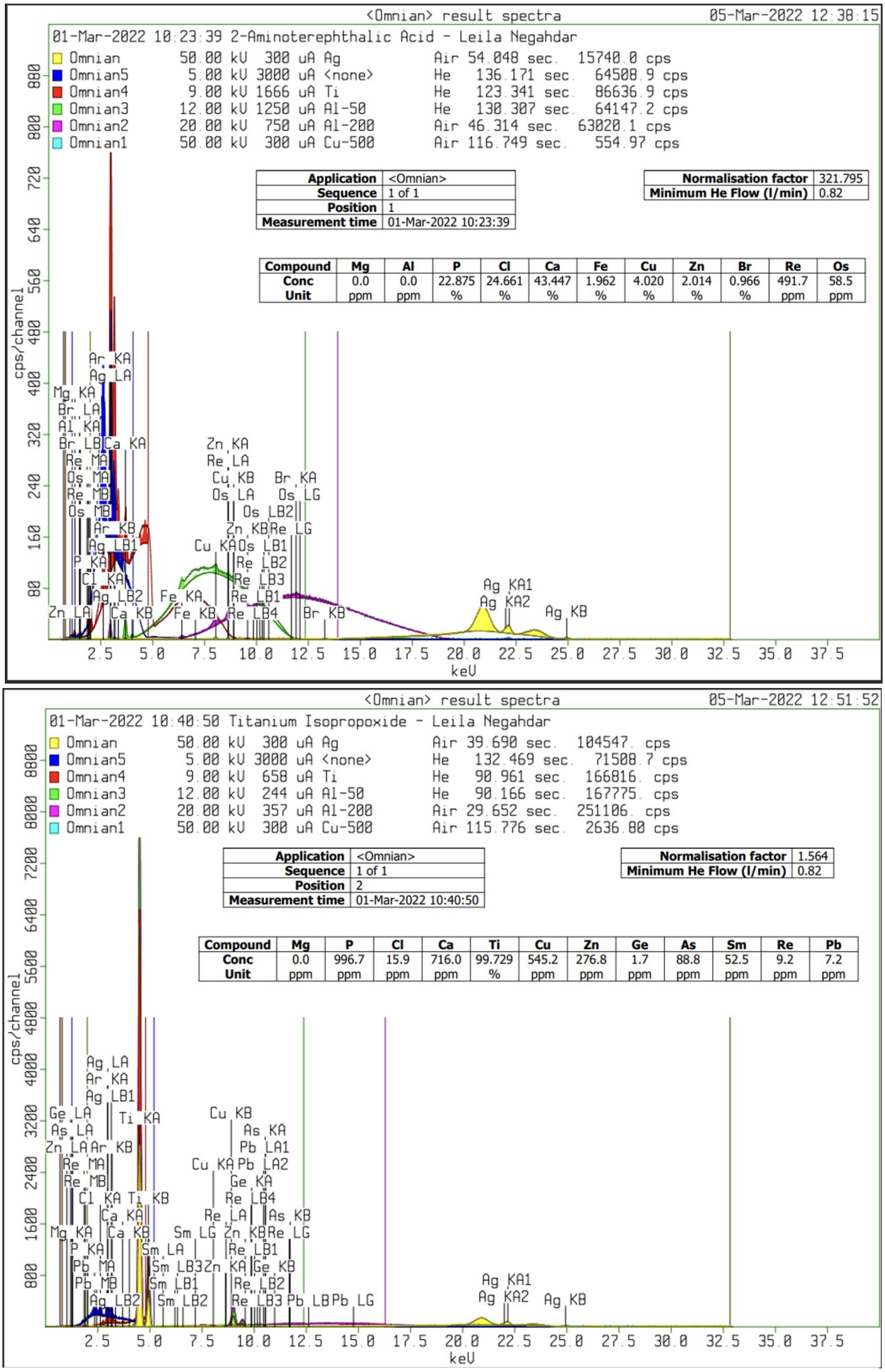

SF.5: XRF Spectra of the starting materials for MOF, MIL-125-NH$_2$. Note the presence of metal impurities that affect the EPR measurements

S.1.3.4 Diffuse Reflectance Infrared Fourier Transform Spectroscopy (DRIFTS)

In situ Diffuse Reflectance Infrared Fourier Transform Spectroscopy (DRIFTS) experiments were conducted on Agilent Cary 600 series spectrometer equipped with Harrick Praying Mantis reaction cell. The gas inlet of the cell was directly connected to a flow system equipped with mass flow controllers and a temperature controller. The cell outlet was connected to the mass spectrometer Hiden QGA MS. In each experiment, 20 mg of catalyst powder was placed in the cell. Before reaction, the KBr background was collected in presence of CO_2 which was flowing through bubbler. 64 scans were collected per spectrum with a spectral resolution of 4 cm^{-1} and in the spectral range of

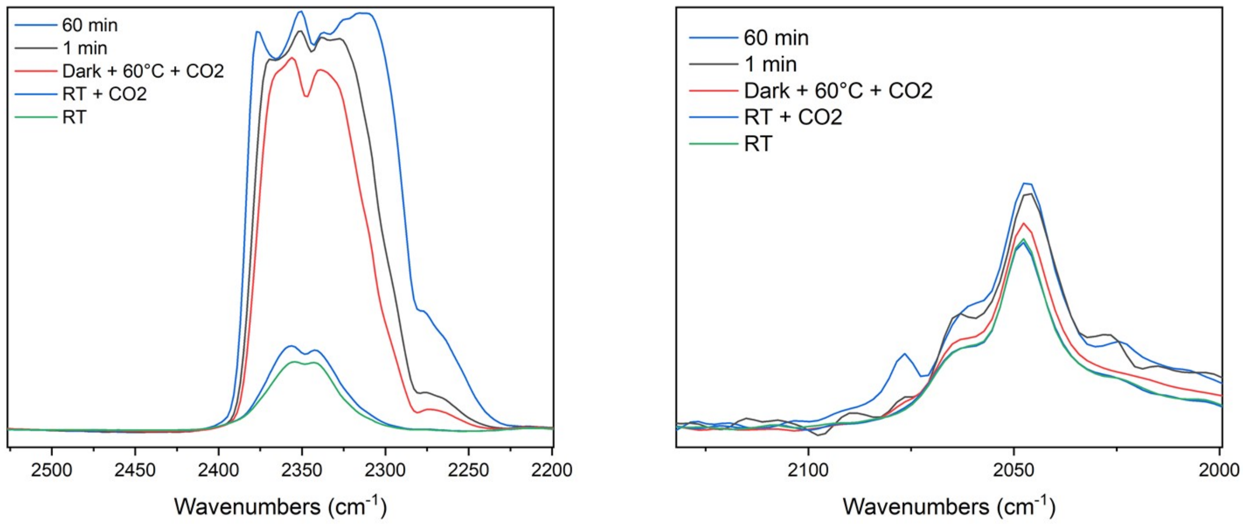

SF 6: MOF-derived TiO_2 DRIFT spectra

4000-400 cm^{-1}.

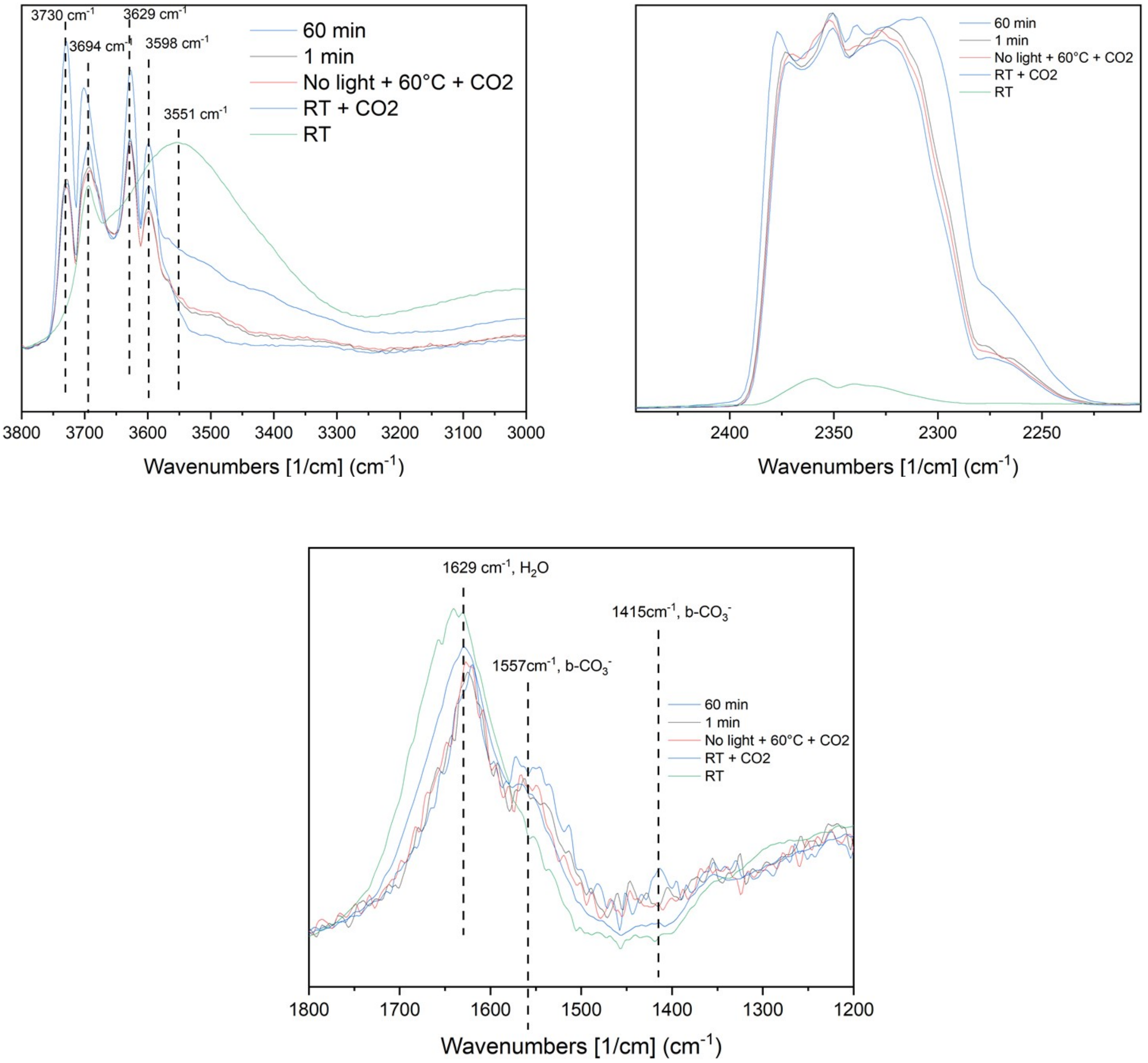

SF 7: P25 DRIFT spectra

S2.1 Design of Experiment

A two-level full-factorial experimental design with three central points was used to systematically investigate the experimental space shown in Table 1 (Main text). To avoid systematic bias and overrating from the size of the parameter values, the values used for evaluating the design were normalised using the '*normalize*' function in MATLAB. MATLAB was also used to estimate: the fitted coefficient values; determine the *p*-values and plot the models and data. The experimental design results were used to fit the function shown by equation (1):

$$Y = \beta_0 + \beta_1 X_1 + \beta_2 X_2 + \beta_3 X_3 + \beta_{12} X_{12} + \beta_{13} X_{13} + \beta_{23} X_{23} + \beta_{123} X_{123} \quad (1)$$

Where Y is the cumulative production of either CO or CH_4; β_0 is the intercept term; X_1, X_2 and X_3 are irradiance, temperature and partial pressure of H_2O respectively; X_{12}, X_{13}, and X_{23} are the two-way interaction terms; X_{123} is the three-way interaction term; β_1, β_2, and β_3 are the coefficients estimated for the impact of irradiance, temperature and partial pressure of H_2O respectively; β_{12}, β_{13}, and β_{23} are

the coefficients estimated for the two-way interactions between the respective parameters ; β_{123} is the three-way interaction term between the parameters.

Using the matrix populated by X values (Table S1) and either cumulative production (CH_4 or CO) as the response Y in equation (1) the coefficients, β, were estimated using by linear regression using a QR decomposition algorithm (*fitlm* function) in MATLAB (Table S2). The *p*-values were then determined using the MATLAB *fitlm* function call. With a 95% confidence, *p*-values less than 0.05 indicated that the coefficient was not equal to zero and therefore its associated parameter had a statistically significant impact on CH_4 or CO cumulative production.

Table S1: Two level full-factorial design with three central points used for experimental settings (X_1, X_2 and X_3) as irradiance, temperature and partial pressure of H_2O respectively. The cumulative production (Y_{CH4} and Y_{CO}) for both CH_4 and CO respectively

Exp. Name	X_1 Irrad	X_2 Temp	X_3 P_{H2O}	X_{12} Irrad.Temp	X_{13} Irrad.P_{H2O}	X_{23} Temp.P_{H2O}	X_{123} Irrad.Temp.P_{H2O}	Y_{CH4} (μmolcm^{-2})	Y_{CO} (μmolcm^{-2})
Exp1	0.00	$4.79x10^{-2}$	$1.75x10^{-1}$	0.00	0.00	$6.69x10^{-2}$	0.00	$1.90x10^{-4}$	$1.17x10^{-2}$
Exp2	$2.67x10^{-2}$	0.00	$8.50x10^{-1}$	$1.60x10^{-3}$	$1.81x10^{-1}$	$2.73x10^{-1}$	$9.12x10^{-2}$	$1.55x10^{-4}$	$7.20x10^{-3}$
Exp3	$6.70x10^{-3}$	$9.62x10^{-1}$	$1.87x10^{-1}$	$2.47x10^{-1}$	$7.80x10^{-3}$	$5.61x10^{-1}$	$1.91x10^{-1}$	$6.21x10^{-4}$	$2.20x10^{-2}$
Exp4	$6.66x10^{-3}$	1.00	1.00	$2.56x10^{-1}$	$1.98x10^{-1}$	1.00	$3.58x10^{-1}$	$3.01x10^{-4}$	$2.28x10^{-2}$
Exp5	1.00	$3.72x10^{-2}$	0.00	$5.41X10^{-1}$	$6.49X10^{-1}$	0.00	$3.66X10^{-1}$	$1.75X10^{-4}$	$1.44X10^{-2}$
Exp6	$9.26x10^{-1}$	$3.19x10^{-2}$	$8.87x10^{-1}$	$4.98x10^{-1}$	1.00	$3.06x10^{-1}$	$5.63x10^{-1}$	$4.44x10^{-4}$	$9.00x10^{-3}$
Exp7	$9.40x10^{-1}$	$9.89x10^{-1}$	$8.75x10^{-2}$	1.00	$6.48x10^{-1}$	$5.24x10^{-1}$	$7.30x10^{-1}$	$7.81x10^{-4}$	$2.87x10^{-2}$
Exp8	$9.13x10^{-1}$	$9.89x10^{-1}$	$8.50x10^{-1}$	$9.78x10^{-1}$	$9.71x10^{-1}$	$9.15x10^{-1}$	1.00	$4.79x10^{-4}$	$2.53x10^{-2}$
Exp9	$5.06x10^{-1}$	$4.57x10^{-1}$	$4.75x10^{-1}$	$4.40x10^{-1}$	$4.76x10^{-1}$	$4.11x10^{-1}$	$4.09x10^{-1}$	$4.49x10^{-4}$	$1.61x10^{-2}$
Exp10	$5.00x10^{-1}$	$3.98x10^{-1}$	$5.62x10^{-1}$	$4.13x10^{-1}$	$5.01x10^{-1}$	$4.13x10^{-1}$	$4.06x10^{-1}$	$4.52x10^{-4}$	$2.10x10^{-2}$
Exp11	$5.00x10^{-1}$	$4.52x10^{-1}$	$6.12x10^{-1}$	$4.34x10^{-1}$	$5.19x10^{-1}$	$4.66x10^{-1}$	$4.36x10^{-1}$	$2.09x10^{-4}$	$1.83x10^{-2}$

Through this study it was found that for both CO and CH_4 production, none of the interaction terms were statistically significant and the model could be simplified to only include the main effects of irradiance (X_1), temperature (X_2) and partial pressure of H_2O (X_3), shown in (S1)

$$Y = \beta_0 + \beta_1 X_1 + \beta_2 X_2 + \beta_3 X_3 \quad (2)$$

Table S2: Coefficient values estimated for fitting model (2) and their respective p-values on CH4 and CO cumulative production. Blue values indicate statistically significant impacts.

**Regression results for CH_4 cumulative production**		
**Parameter coefficient**	**Value estimated**	***p*-value**
β_1	$1.42x10^{-4}$	$2.63x10^{-1}$
β_2	$3.22x10^{-4}$	$2.48x10^{-2}$
β_3	$-1.17x10^{-4}$	$4.18x10^{-1}$
**Regression results for CO cumulative production**		
**Parameter coefficient**	**Value estimated**	***p*-value**
β_1	$3.21x10^{-3}$	$1.14x10^{-1}$
β_2	$1.48x10^{-2}$	$5.62x10^{-5}$
β_3	$-3.49x10^{-3}$	$1.37x10^{-1}$

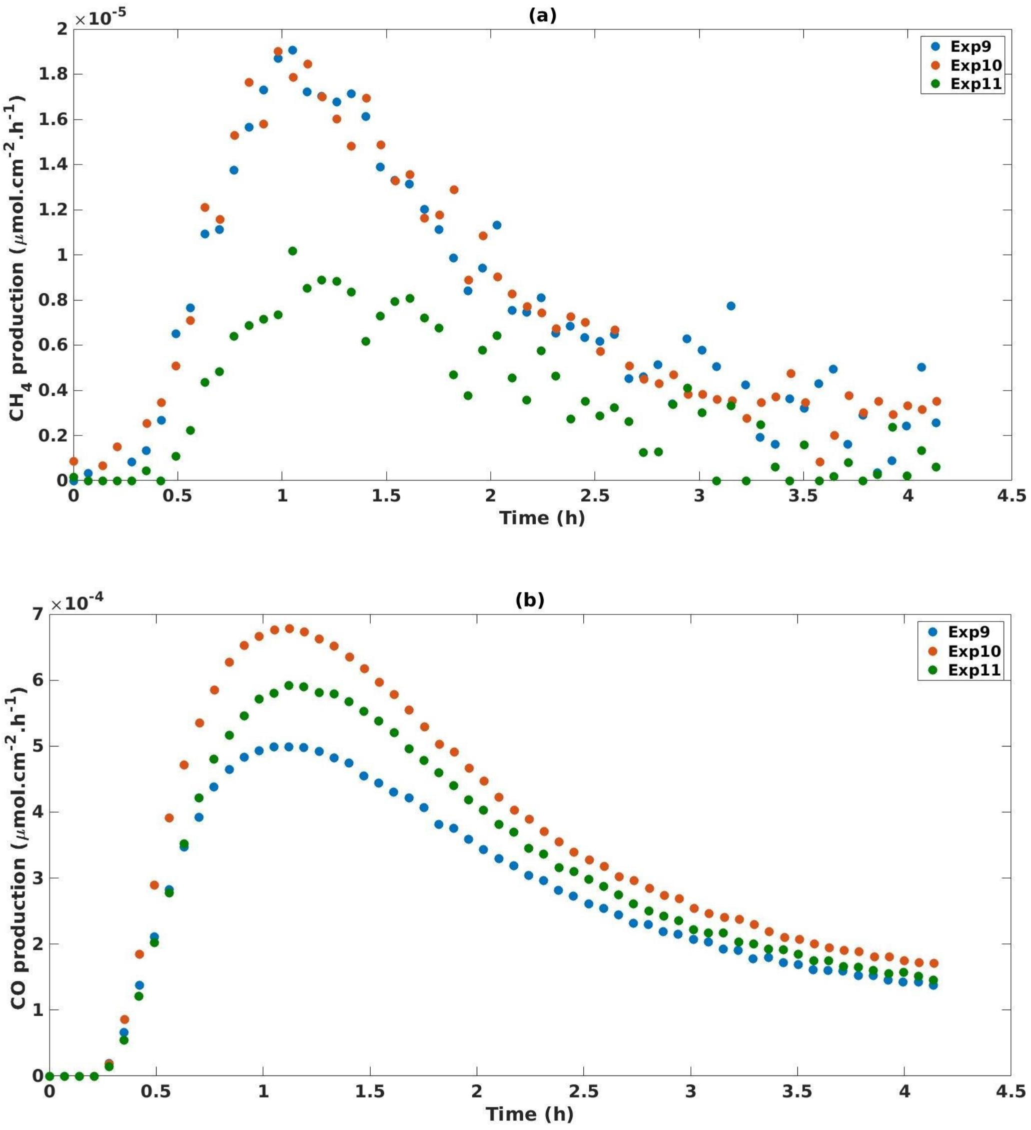

SF 8 *:(a) CH_4 and (b) CO production using central point experimental settings from the full-factorial design*

S2.2 Reproducibility

Consistency shown in **SF 8** indicate a high level of reproducibility in the central points of the DOE.

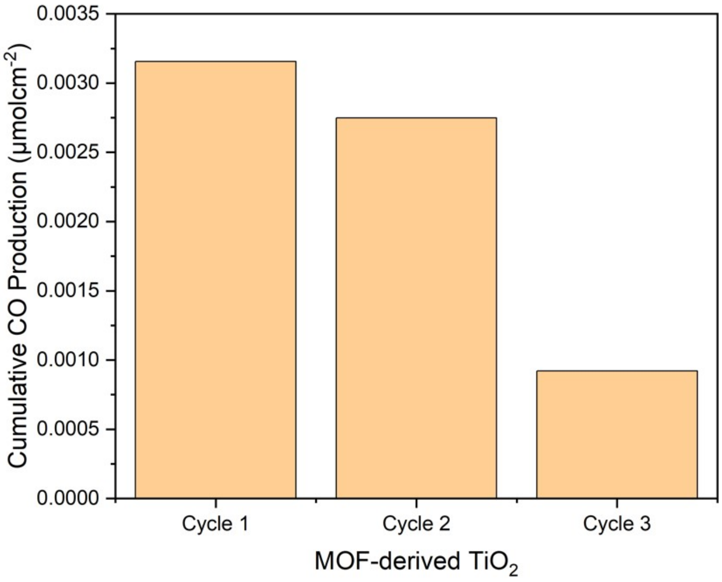

***SF 9**: Cycle tests of for MOF-derived TiO_2*

S3 Referenced Work

Table S3: Comparison of MOF-derived TiO_2 to other state-of-the-art TiO_2 based photocatalysts

Photocatalyst	Light source	Duration of Irradiance	Continuous or Batch	Conditions	Products	Ref
TiO_2-in-MIL-101-Cr	300 W Xe lamp (Beijing PerfectLight)	60 h	Batch, sampled every 1 hour	45 °C Reactor Temp	1.1 mmolg^{-1}h^{-1} CH_4<br>11 mmolg^{-1}h^{-1} CO	29
Pt-TiO_2	400 W Xe Lamp Light irradiance 19.6 mW/cm^2	8 h	Continuous	UV range (250–388 nm)	1361 µmolg^{-1}h^{-1} CH_4<br>179 µmolg^{-1}h^{-1} CO	30
DPSNs@TiO_2@Au	300 W Xe lamp (PLS-SXE300)	4 h	Batch, sampled every 1 h	20 °C reactor Temp	35.25 µmolg^{-1}h^{-1} CH_4<br>15.27 µmolg^{-1}h^{-1} CO	31
$CpRu_{0.6}/TiO_2$	300W Xe lamp	16 h	Batch, sampled every 4 hours	Room Temperature 420 nm cut-off filter	44.0 µLg^{-1}h^{-1} CH_4	51
SBNT-HR-0.5	300 W Xe lamp	4 h	Batch, sampled every 1 h	20 °C Reactor temp	17.11 µmolg^{-1}h^{-1} CO	52
TiO_2@50Cu	300 W Xe arc lamp (CEL-HXF300, Beijing China Education Au-light Co., Ltd)	3 h	Batch, sampled every 1 h	20 °C Reactor temp	4.3 µmolg^{-1}h^{-1} CH_4<br>23.25 µmolg^{-1}h^{-1} CO	53
H-TiO_2@50Cu					0.94 µmolg^{-1}h^{-1} CH_4<br>10.58 µmolg^{-1}h^{-1} CO	
MOF-derived TiO_2	OmniCure S2000 (300 – 600 nm)	4 h	Continuous	56 °C Reactor temp 2850 W.m^{-2}	0.1 µmol g^{-1} h^{-1} CH_4<br>2.6 µmol g^{-1} h^{-1} CO	This work
